# Effects of Perforation on Rigid PU Foam Plates: Acoustic and Mechanical Properties

**DOI:** 10.3390/ma9121000

**Published:** 2016-12-09

**Authors:** Jia-Horng Lin, Yu-Chun Chuang, Ting-Ting Li, Chen-Hung Huang, Chien-Lin Huang, Yueh-Sheng Chen, Ching-Wen Lou

**Affiliations:** 1 Department of Chemistry and Chemical Engineering, Minjiang University, Fuzhou 350108, China; jhlin@fcu.edu.tw; 2 Laboratory of Fiber Application and Manufacturing, Department of Fiber and Composite Materials, Feng Chia University, Taichung 40724, Taiwan; p0200108@fcu.edu.tw; 3 School of Chinese Medicine, China Medical University, Taichung 40402, Taiwan; 4 Department of Fashion Design, Asia University, Taichung 41354, Taiwan; 5 Innovation Platform of Intelligent and Energy-Saving Textiles, School of Textiles, Tianjin Polytechnic University, Tianjin 300387, China; tingtingli@tjpu.edu.cn; 6 Department of Aerospace and Systems Engineering, Feng Chia University, Taichung 40724, Taiwan; chhuang@fcuoa.fcu.edu.tw; 7 Department of Fiber and Composite Materials, Feng Chia University, Taichung 40724, Taiwan; clhuang@mail.fcu.edu.tw; 8 Department of Biomedical Imaging and Radiological Science, China Medical University, Taichung 40402, Taiwan; yuehsc@mail.cmu.edu.tw; 9 Graduate Institute of Biotechnology and Biomedical Engineering, Central Taiwan University of Science and Technology, Taichung 40601, Taiwan

**Keywords:** perforation process, polyurethane (PU) foam, mechanical properties, sound absorption coefficient

## Abstract

Factories today are equipped with diverse mechanical equipment in response to rapid technological and industrial developments. Industrial areas located near residential neighborhoods cause massive environmental problems. In particular, noise pollution results in physical and psychological discomfort, and is a seen as invisible and inevitable problem. Thus, noise reduction is a critical and urgent matter. In this study, rigid polyurethane (PU) foam plates undergo perforation using a tapping machine. The mechanical and acoustic properties of these perforated plates as related to perforation rate and perforation depth are evaluated in terms of compression strength, drop-weight impact strength, and sound absorption coefficient. Experimental results indicate that applying the perforation process endows the rigid PU foaming plates with greater load absorption and better sound absorption at medium and high frequencies.

## 1. Introduction

Many factories are situated near residential districts and cause environmental problems, such as noise pollution. The operation of numerous machines contributes to a large amount of noise. Moreover, noise can be magnified through multi-reflection caused by the design of the walls and ceiling in a workplace, thereby jeopardizing the residents’ quality of living. In physics, noise is defined as sound waves that have irregular frequencies and produce an unpleasant, irritating, or disturbing sound. Noise creates dysphoria and poses hazards to human health [1,2]. Noise has different attributes according to its distribution of energy and frequency. High frequency noise is estimated to start from 1500 Hz, which refers to sharp sounds with short sound waves. Therefore, high frequency noise can be attenuated when obstacles exist. Low frequency noise is below 500 Hz, and its acoustic character spreads via structural transmission, airborne transmission, and standing waves. Moreover, low frequency noise is not subject to attenuation during spread and thus causes the most damage to human health [3].

People who live in highly industrialized cities have gradually become aware of noise hazards. To decrease the damage to the natural environment and people’s health, commonly used noise controls are segregation of noise sources and propagation path, and protection of recipients of noise. In particular, sound absorption and insulation materials are combined to surround a noise source, which will change or isolate the propagation path to attain noise reduction [4,5]. Sound absorption and sound insulation are commonly used to reduce noise. Sound absorption applies a mechanism whereby sound waves are primarily absorbed by materials, while a lesser amount of sound waves are reflected via the surface and refracted via the interior. Generally, sound absorbing materials have a porous structure, and some sound waves can be reflected by their surface, while some others are multiple-refracted in their interior, thereby dissipating the sound energy. Sound absorbing materials include glass fiber [6], artificial fiber [7,8,9], and natural fibers [10,11], and sound insulating materials are foam materials, such as phenolic foam and polyurethane foam [12,13,14].

In sound insulation, noise sources are contained or reflected using sound insulating materials to reduce the transmission or volume of noise. Usually, materials do not exhibit sound absorption and sound insulation at the same time. A material that combines these two features can exhibit a greater acoustic effect [4], which has been the goal of many recent studies. Kino et al. examined the vibration of cell-contained PU films in an impedance tube to determine the influence on the acoustic properties [15]. Zhang et al. discovered that an interconnected structure of appropriate porous mediums helped to improve their sound absorbing performance, thereby profiling the acoustic efficiency of PU foam. The test results showed that the cell sizes and interconnected pores pertain to the sound absorbing efficiency of materials [13]. Ren et al. hypothesized that increasing the frictional force between air and the cells in PU foam is the most efficient method to improve sound absorption. Their test results indicated that the sound absorption of PU foam significantly improved as a result of the increasing flow resistance of samples against sound waves [16]. Moreover, Tiuc et al. examined how the perforation of sound-absorbing materials is correlated with the sound absorption coefficient and found that perforation improved the sound absorption coefficient when the frequency of sound is below 1100 Hz. This perforated sound-absorbing material is suitable for use in industry, transportation, and air transportation [17].

Conventional perforated panels and micro-perforated panels have been commonly used to control noise or as a protective surface for porous materials. The acoustic efficacy of perforated panels is dependent on perforation rate, pore size, thickness of the plate, flow resistance, and the installation condition [18,19]. Micro-perforated panels (MPP) were developed by Maa in 1975 as a result of the shortage of porous fiber materials. MPP has abundant resource materials, including cardboards, plastic, and metal plates. Unlike conventional perforated panels that are made of materials with a pore size at the millimeter or centimeter level, MPP has a pore size that is at sub-millimeter and does not require any porous fiber materials. In addition, MPP is equipped with a resonance chamber behind it, thereby obtaining sound absorption efficacy [20,21,22,23]. Compared with conventional perforated panels, micro-perforated panels have a higher sound absorption coefficient and a wider range of sound absorbing bandwidth [24]. PU foam, which is one of the most popular polymer materials, has an efficient and flexible production technique, and its density, strength, and stiffness can be changed to suit different applications. PU foams come in rigid and flexible types [25,26]. Rigid PU foam has a closed-cell structure, good thermal insulation, a light weight, specific strength, efficient construction, sound and electric insulation, and solvent resistance, and it is shockproof. Thus, rigid PU foam has been commonly used as the filler for the insulation/buffer layer of refrigeration cabinets, as well as the inner layer of buildings’ walls [27,28]. By contrast, flexible PU foams have an open-cell structure, high strength, high resiliency, and efficient processing; as such, they are suitable for use in buffering and sound absorbing materials [29]. In this study, PU foam plates are composed of isocyanate, and polyol and are perforated with a pore diameter of 1 mm. PU foams are composed of pores that have different characteristics from that of the pores formed by perforation. These pore structures also dissipate and attenuate sound waves through different sound absorption mechanisms, where PU foam uses a cell elastic resonance, and a perforated sample uses damping attenuation. Therefore, a compound structure that applies both mechanisms can effectively decrease noise and has thus become a popular trend for sound absorbent materials [30]. The samples are tested for compression, drop-weight impact, and sound absorption coefficient; the average sound absorption coefficient is computed, thereby characterizing the mechanical and acoustic properties.

## 2. Experimental Section

### 2.1. Materials

PU foam solvent, which is composed of polyol foaming agent and isocyanate (MDI) hardener, is provided by Zhongxing Chemical, Taichung, Taiwan, R.O.C.

### 2.2. Methods

**Figure 1:**
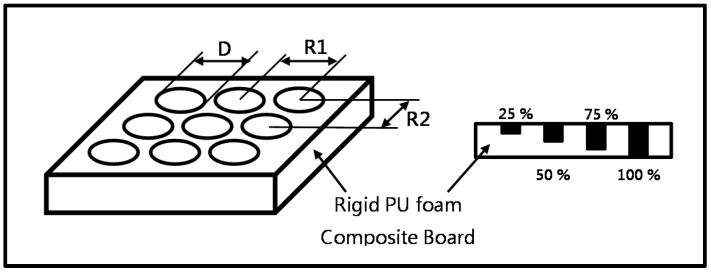
Schematic illustration of pore locations and perforation depths. *D* is the diameter of pores. *R*1 is equal to *R*2. The perforation rate and corresponding *R*1 are 1% (9 mm), 3% (5 mm), and 5% (4 mm).

PU foam is composed of polyol and MDI at different ratios, and the optimal ratio is determined to be 1:1. Polyol and MDI are blended and mixed at a speed of 600 rpm for 30 s. The mixture is poured into a mold with dimensions of 300 mm × 300 mm × 20 mm to achieve a specific volume density. The mold is cooled at room temperature for 120 min, while the mixture is solidified to form PU foam plates. The specific density of PU foam plates is 60 kg/m^3^. The plates are perforated, creating pores with a diameter of 1 mm. The perforation rates are 0%, 1%, 3%, and 5%, the perforation depths are 25%, 50%, and 75%, and the plates’ thickness is 100%. The PU plates that have different perforation conditions are tested for drop-weight impact and sound absorption coefficient. Figure 1 shows the perforation conditions. The perforation rate and pore location are computed using Equation (1).

(1) $$\frac{D^{2}\times 78.50}{R1\times R2}$$

where *D* refers to the diameter of the pore (1 mm), and *R*1/*R*2 refers to the distance between the center of two adjacent pores.

### 2.3. Tests

#### 2.3.1. Compression

As specified in ASTM D1621-16, the standard test method for compressive properties of rigid cellular plastics, the sample is cut into 50 mm × 50 mm × 20 mm pieces. The testing speed is 2 mm/min. Eight samples for each specification are used. The samples are compressed into 25% thickness of the original.

#### 2.3.2. Drop-Weight Impact

Drop-weight impact testing is conducted according to ASTM D4168-95 (2015). The impactor has a weight of 8.5 kg, and the impact load is 9000 N. Six samples for each specification are used for this test. The impactor is released from a height of 4 cm above the samples and vertically hit the surface of 100 mm × 100 mm samples. The residual load is used to characterize the cushioning property of PU foam plates.

#### 2.3.3. Sound Absorption Coefficient

**Figure 2:**
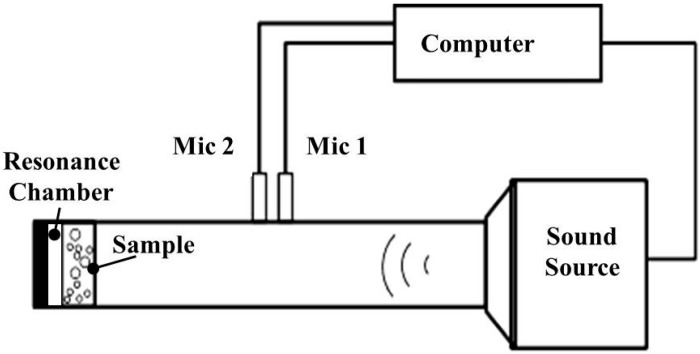
Schematic illustration of the sound absorption coefficient tester.

As specified in ASTM E1050-12, a two-microphone impedance tube (Automotive Research & Testing Center, Taipei, Taiwan) is used to measure the sound absorption coefficient of the PU foam plates at a frequency between 125 and 4000 Hz (Figure 2). Samples are cut into circular sections with a diameter of 38 mm. Three samples for each specification are used for this test. During the test, samples are placed in the tube, and the distance from the end of the tube to the sample is varied at 0, 5, 10, 15, and 20 mm. This particular parameter is hereafter referred to as resonance chamber size. When the samples are perforated at a depth of 100%, the tunnels formed in the PU foam plates and the sealed resonance chamber compose the Helholtz resonance structure [31], thereby attaining different acoustic effects according to the cell and compression resonance. Sound absorption average (SAA) is determined by the arithmetic average of twelve one-third octave frequency band acoustic absorption coefficient values from 1500 through 4000 Hz. As samples are prepared according to the diameter of the two-microphone impedance tube, more precise results can be obtained with further tests (e.g., anechoic chamber) on samples in end-use dimensions. Like sound absorption coefficient, noise reduction coefficient (NRC) is also an overall evaluation of the sound absorption ability of a specified material within a closed space and is the mean of the sound absorption coefficients at 250, 500, 1000, and 2000 Hz.

#### 2.3.4. Determination of Sound Absorption Coeficient

Sound absorption coefficient (α) is computed based on sound reflection coefficient (*r*) of normal incidence as well as transfer function (*H*_12_) as specified in standard of BS EN ISO 10534-2:2001.

(2) $$\mathrm{Sound}\;\mathrm{reflection}\;\mathrm{coefficient}\;\left(r\right)=\frac{H_{12}-H_{I}}{H_{R}-H_{12}}\;e^{2jk_{0}x_{1}}$$

where *H*_12_ is the acoustic transfer function, *H_I_* is the transfer function of incident wave, *H_R_* is reflected wave, and *k*_0_ is the wave number.

(3) $$\mathrm{Wave}\;\mathrm{number}\;\left(K_{0}\right)=\frac{2\pi f}{C_{0}}$$

where *f* is the frequency and *C*_0_ is the speed of sound.

(4) $$\mathrm{Transfer}\;\mathrm{function}\;(H_{12})=\frac{p_{2}}{p_{1}}=\frac{\;e^{jk_{0}x_{2}}+r\;e^{-jk_{0}x_{2}}}{\;e^{jk_{0}x_{1}}+r\;e^{-jk_{0}x_{1}}}$$

where *p*_1_ and *p*_2_ are the measured acoustic pressure of the two microphones, and *x*_1_ and *x*_2_ are the distance between the reference plane (sample position of *x* = 0) and the two microsphones.

(5) $$\mathrm{Trasfer}\;\mathrm{Function}\;\mathrm{of}\;\mathrm{Incident}\;\mathrm{Waves}\;(H_{I})=\;e^{-jk_{0}\left(x_{1}-x_{2}\right)}$$

(6) $$\mathrm{Transfer}\;\mathrm{function}\;\mathrm{of}\;\mathrm{reflective}\;\mathrm{waves}\;(H_{R})=\;e^{jk_{0}\left(x_{1}-x_{2}\right)}$$

(7) $$\mathrm{Sound}\;\mathrm{absorption}\;\mathrm{coefficient}\;\left(\alpha \right)=1-{\left|r\right|}^{2}=1-r_{r}^{2}-r_{i}^{2}$$

where $r_{r}$ is the real component and $r_{i}$ is the imaginary component.

#### 2.3.5. Statistical Analyses

One-way ANOVA of SPSS is used for statistical analyses of the measured data. The α level is set to be 5% with a confidence interval of 95%. “*” means *p* < 0.05, indicating difference, and “**” means *p* < 0.01, indicating the signficant difference.

## 3. Results and Discussion

### 3.1. Compression

**Figure 3:**
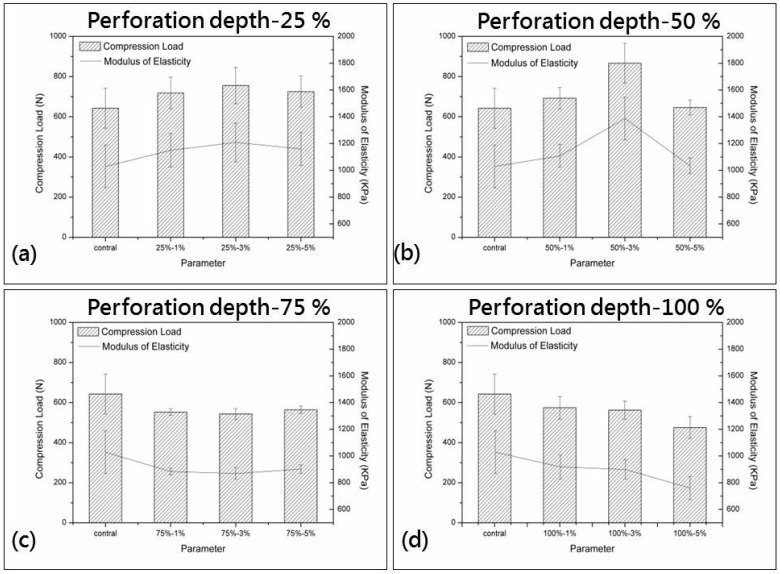
Compression load of PU foam plates in relation to perforation depths of (**a**) 25%; (**b**) 50%; (**c**) 75%; and (**d**) 100%.

The compression load of the rigid PU foam plates is tested, and the data are shown in Figure 3. When the PU foam plates are compressed, the constituent pores first exhibit elastic deformation and then start to collapse, deform, or squeeze to disperse the load, as the plastic deformation does not dissipate the load [32]. Figure 3 shows the compression load of the plates in relation to the differing combinations of perforation rate and perforation depth. Test results show that compared with non-perforated samples, plates that are perforated at depths of 25% and 50% have a greater compression load regardless of the perforation rate. Moreover, increasing the perforation rate first increases and then decreases the compression load of the plates. Conversely, plates that are perforated at depths of 75% and 100% have a lower compression load than the non-perforated samples. During the test, a perforation depth that is less than 50% allows the compression load to be dispersed along the pores, and these samples exhibit satisfactory elastic deformation. Moreover, the undamaged areas of the perforated samples create a supportive structure. Thus, a perforation depth that is lower than 50% has a positive influence on the compression load of the PU foam plates. An excessive perforation depth (higher than 50%) decreases the supportive structure of the PU foam plates, thereby decreasing the compression load. With the use of one-way ANOVA, the compression data of perforated PU foam plates are compared in terms of perforation rate and perforation depth. No significant difference is found in the compression in relation to perforation rate (*p* = 0.526), whereas a significant difference is found in the compression in relation to perforation depth (* *p* = 0.026), as seen in Table 1. In sum, the optimal compression load of 866.5 N occurs when the plates are made with a perforation depth of 50% and a perforation rate of 3%.

### 3.2. Drop-Weight Impact

**Figure 4:**
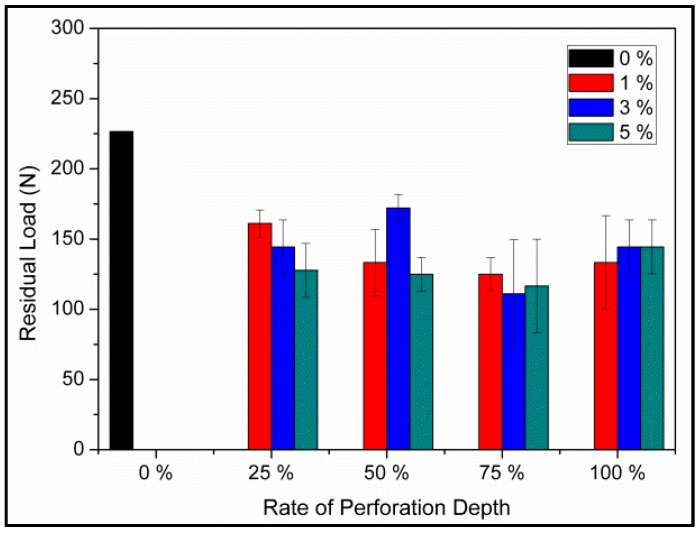
Residual load of PU foam plates in relation to perforation rates (0%, 1%, 3%, and 5%) and perforation depths (25%, 50%, 75%, and 100%).

**Figure 5:**
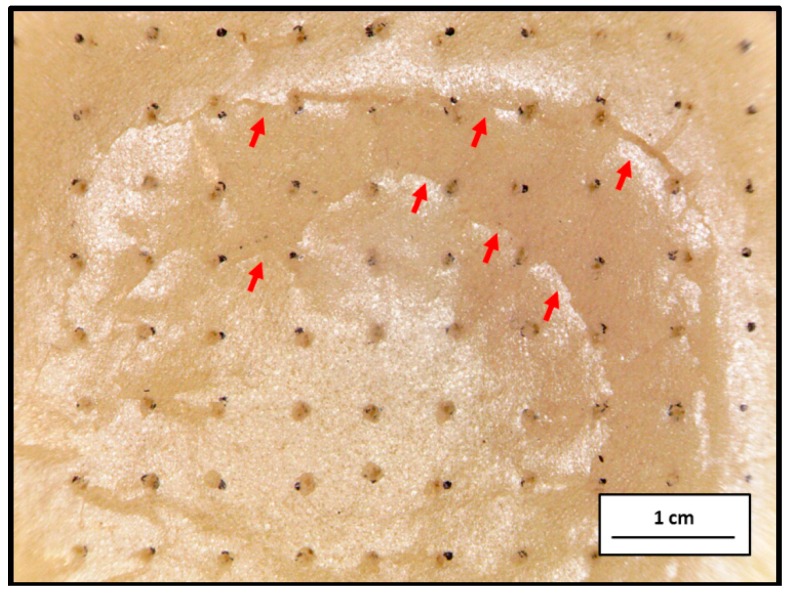
Image of cracks in the PU foam plate.

Figure 4 shows the residual load of PU foam plates in relation to different perforation rates of 0%, 1%, 3%, and 5% and perforation depths of 25%, 50%, 75%, and 100%. The test results indicate that the perforated PU foam plates exhibit lower residual load than the non-perforated ones. The structure of the plates is damaged as a result of undergoing perforation; as a result, the plates become less supportive. A comparison with the non-perforated samples indicates that the perforated PU foam plates exhibit greater extrusion deformation and collapse of the pores caused by perforation when an instant impact is applied. Furthermore, the area between pores caused by perforation also generates cracks, which dissipates more of the impact energy (Figure 5). In addition, variations in perforation rate and perforation depth do not lead to a significant difference in the residual load of the perforated samples. As a result, perforated PU foam plates exhibit higher absorption characteristics of impact energy in comparison to the non-perforated samples. In particular, the lowest residual impact load of 110.4 N occurs when the perforated plates are made with a perforation depth of 75% and a perforation rate of 3% during the 9000 N drop-weight impact test. ANOVA results indicate that neither perforation rate nor perforation depth pertains to the drop-weight impact behavior of the PU foam plates (*p* > 0.05).

### 3.3. Sound Absorption Coefficient

**Figure 6:**
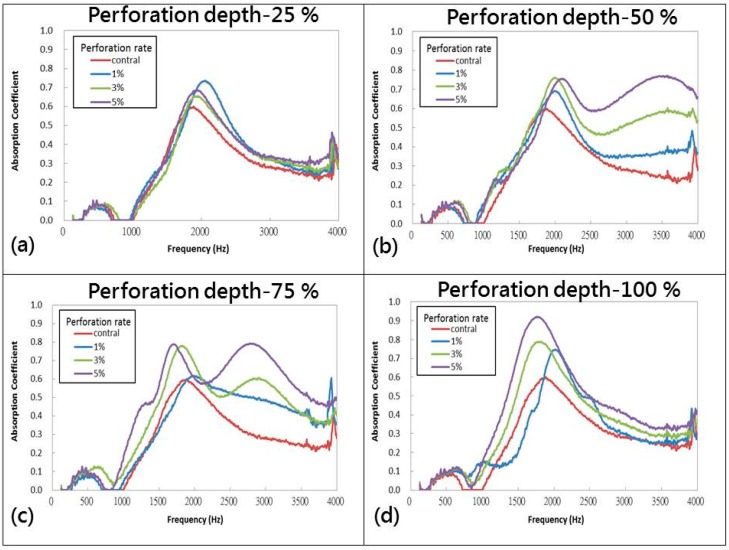
Sound absorption coefficient of PU foam plates in relation to perforation depths of (**a**) 25%; (**b**) 50%; (**c**) 75%; and (**d**) 100%.

Figure 6 shows the sound absorption coefficient of PU foam plates in relation to different perforation rates of 0%, 1%, 3%, and 5% and perforation depths of 25%, 50%, 75%, and 100%. The test results indicate that the perforated plates show a slightly improved sound absorbing effect on sound waves at a frequency of 125–4000 Hz. The characteristic peak is 2000 Hz for samples with perforation depths of 25%, 50%, and 100%, and it is 1800 Hz for samples with a perforation depth of 75%, that is, the majority of the samples exhibit optimal sound absorption effect against sound waves at 2000 Hz. In particular, using perforation depths of 50% and 75% improves the sound absorbing effect for sounds at a frequency of 2500 Hz. PU foam has a closed-cell structure, and the cells are not interconnected. Primarily, PU foam dissipates sound energy via elastic compression and vibration of the cells. Incident waves that approach PU foam plates are attenuated because of the loss of sound energy; some of the waves are reflected, while some are dissipated via the vibration between sound energy and the plates. As such, only a certain amount of sound waves enter the plates through the pores. Moreover, the surface of perforated PU foam plates allows for easy access of sound waves to the interior, thereby decreasing the extent of sound reflection. The air in the pores then forms a relative velocity against the incident sound waves. The abrasion between high speed air molecules and the stagnant air molecules, as well as the friction between sound waves and rough surface of pores, contributes to the transformation of the kinetic energy into heat energy, thereby achieving sound absorption.

When the sound waves have a frequency higher than 2500 Hz, PU foam plates with perforation depths of 50% and 75% and a high perforation rate exhibit a greater sound absorption effect. Conversely, the use of a perforation depth of 100% results in the penetration of sound waves through the plates, thereby causing a relatively lower sound absorption effect. Table 2 shows the NRC, SAA, corresponding frequency, and sound absorption coefficient (α_max_) of plates in relation to perforation depth and perforation rate. Specifically, the optimal sound absorption effect occurs when the plates are made with a perforation depth of 50% and perforation rate of 3%, and when the incident sound waves have a frequency of 125–4000 Hz.

### 3.4. Sound Absorption Coefficient in Relation to Resonance Chamber Size

**Figure 7:**
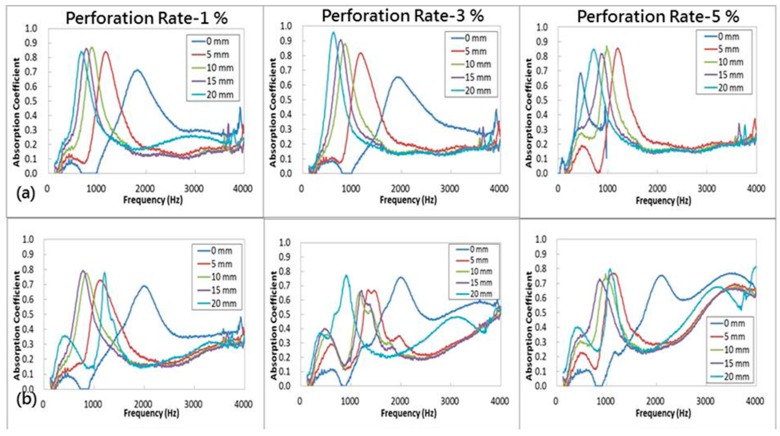

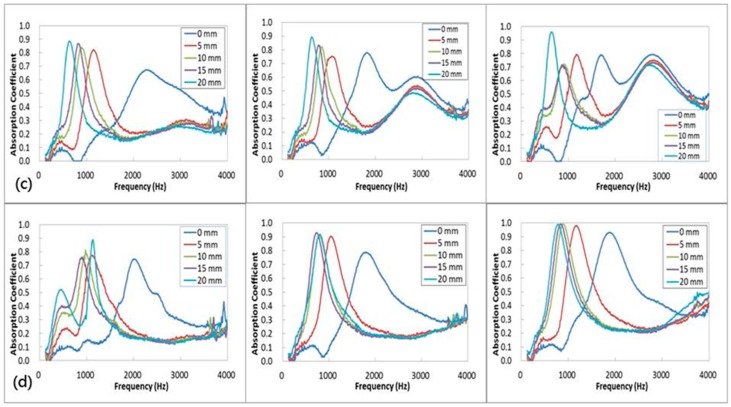
Sound absorption coefficient of PU foam plates in relation to perforation depths of (**a**) 25%; (**b**) 50%; (**c**) 75%; and (**d**) 100% and the resonance chamber size (0, 5, 10, 15, and 20 mm).

Figure 7 shows the influence of resonance chamber size on the sound absorption of PU foam plates made under different perforation conditions. During the test, samples are placed in the tube, and the distance from the end of tube to the sample is varied at 0, 5, 10, 15, and 20 mm; this distance is the parameter (resonance chamber size). Moreover, when the samples are perforated at a depth of 100%, the tunnels formed in the PU foam plates and the sealed resonance chamber compose the Helholtz resonance structure, where the acoustical energy is decreased because of the sound cell resonance and compression resonance. The sound absorption coefficient test results are consistent, that is, increasing the resonance chamber size shifts the characteristic peaks of PU foam plates from 2000 Hz to a lower frequency regardless of the perforation depth. However, when the perforation depth is 50% and 75%, increasing the resonance chamber size does not have any significant influence on the sound absorption effect of the plates at a frequency of 2500 Hz. Sound waves at different frequencies have different characteristics, and thus, they do not exhibit the same dissipation of sound energy. A low frequency corresponds to a long wavelength. Hence, resonance dissipation of sound energy at low frequency is used for sound absorption. High frequency sound waves have short wavelengths and can thus be dissipated using porous materials via the abrasion of air molecules to attain sound absorption. Therefore, a chamber is maintained behind the plates to increase the resonance space, thereby leading to more refraction of sound waves. As a result, the characteristic peak of plates gradually shifts from 2000 Hz to lower frequencies.

The Helmholtz resonator frequently has a resonance frequency (*f*_0_). Materials exhibit greater sound absorption when they are tested with a frequency that is comparable to *f*_0_. Therefore, Equation 8 is used to compute the resonance frequency for samples at a 100% perforation depth to examine the differences between theoretical and practical results shown in Table 3. A comparison between the test results and Figure 7d shows a significant difference when the perforation rate is high, which is ascribed to the constitute PU foam plates. The cell elastic resonance sound absorption and the perforated Helmholtz resonator thus contribute complex acoustic efficacy against sound waves.

(8) $$f_{0}=\frac{c}{2\pi }\sqrt{\frac{P}{\left(t+0.8d\right)L}}$$

where $f_{0}$ is the resonance frequency, *c* is the acoustic velocity, *p* is the perforation rate, *t* is the thickness of perforated plates, *d* is the diameter of pores, and *L* is the resonance chamber size.

In addition, the perforated plates with a perforation depth lower than 100% are not connected with the resonance chamber. Therefore, these samples cannot be considered a Helmholtz resonator, and such perforation helps in decreasing the incident damp over the PU foam’s surface, thereby decreasing the amount of sound waves that are directly reflected. Afterwards, the sound waves enter samples through the pores. The relative velocity between sound waves and stagnant air molecules of the pores compresses the latter at the same time, thus strengthening the air density and pressure. The compressed air molecules then travel toward the pore bottoms, thereby causing more minor resonance vibrations and frictions against the pores. The sound waves are thus confined within the pores and converted into standing waves, and their kinetic energy is converted into thermal energy that dissipates gradually.

## 4. Conclusions

This study investigated perforated rigid PU foam plates that have buffering and sound absorbing effects. The test results showed that the perforated plates exhibit a higher impact energy than the non-perforated plates. The sound absorption of perforated plates slightly increased when the perforation rate increased. Moreover, when the perforation depth is 50% and 75%, the sound absorption of plates at a frequency of 2500 Hz is significantly improved as a result of the increased perforation rate. Specifically, the optimal sound absorption at frequencies of 125–4000 Hz occurs when the plates are made with a perforation depth of 50% and 75%, and a perforation rate of 3%. When the perforated PU foam plates are used with a resonance chamber, they exhibit satisfactory sound absorption property in a frequency range of 500–1000 Hz. The test results provide a valuable reference for perforated PU foam plates in light of different environmental noises. These plates are thus suitable for use as walls and ceilings of factories and buildings.

## Figures and Tables

**Table 1:** One-way ANOVA summary of the compression of PU foam plates as related to perforation depth.

Perforation Depth	Perforation Rate	Compression Load (N)	Modulus of Elasticity (KPa)	CV%	*p* Value
0%	Control	642.90 ± 99.27	1028.63 ± 158.83	15.44	-
1%	25%	718.43 ± 78.58	1149.49 ± 125.72	10.94	*
	50%	692.37 ± 52.70	1107.79 ± 84.32	7.61	*
	75%	552.53 ± 15.66	884.04 ± 25.06	2.83	-
	100%	574.05 ± 56.06	1028.63 ± 158.83	9.77	-
3%	25%	755.27 ± 90.47	1208.43 ± 144.76	11.98	*
	50%	866.49 ± 99.28	1386.39 ± 158.84	11.46	**
	75%	543.03 ± 27.35	868.85 ± 43.76	5.04	-
	100%	562.17 ± 44.44	899.47 ± 71.10	7.90	-
5%	25%	724.97 ± 77.35	1159.95 ± 123.77	10.67	**
	50%	646.12 ± 36.87	1033.79 ± 58.99	5.71	**
	75%	563.92 ± 18.86	902.28 ± 30.17	3.34	*
	100%	475.23 ± 54.18	760.37 ± 86.69	11.40	-

Note: *p* < 0.05 (*) and *p* < 0.01 (**).

**Table 2:** NRC, SAA, corresponding frequency, and sound absorption coefficient of PU foam plates.

Perforation Depth—Perforation Rate	Noise Reduction Coefficient (NRC)	Sound Absorption Average (SAA)	Frequency (Hz) at α_max_	α_max_
control	0.160	0.364	1880	0.60
25%—1%	0.210	0.418	2000	0.73
25%—3%	0.182	0.402	1900	0.65
25%—5%	0.192	0.427	1900	0.68
50%—1%	0.224	0.437	2000	0.69
50%—3%	0.237	0.560	2000	0.76
50%—5%	0.230	0.655	2100	0.76
75%—1%	0.181	0.482	2000	0.62
75%—3%	0.225	0.540	1800	0.78
75%—5%	0.223	0.640	1700	0.79
100%—1%	0.248	0.396	2000	0.75
100%—3%	0.226	0.459	1800	0.79
100%—5%	0.263	0.527	1750	0.92

**Table 3:** Resonance frequency ($f_{0}$) in Hz of perforated plates at a 100% perforation depth.

Perforation Rate (%)	Resonator Thickness (mm)			
	5	10	15	20
1	845 Hz	597 Hz	487 Hz	422 Hz
3	1463 Hz	1035 Hz	845 Hz	731 Hz
5	1889 Hz	1336 Hz	1091 Hz	944 Hz
